# Downregulation of the Transglutaminase 2–NF-κB Inflammatory Axis by a Fusion Protein of Cementoin and Secretory Leukocyte Protease Inhibitor Reduces Corneal Angiogenesis

**DOI:** 10.3390/ijms27073247

**Published:** 2026-04-02

**Authors:** Juan Pablo Salica, María Constanza Potilinski, Gustavo Ortiz, Paulo C. Maffia, Diego Guerrieri, Eduardo Chuluyan, Juan Eduardo Gallo

**Affiliations:** 1 Instituto de Investigaciones en Medicina Traslacional (IIMT), Facultad de Ciencias Biomédicas, Universidad Austral, and CONICET, Pilar, Buenos Aires B1629, Argentina; jp.salica@gmail.com (J.P.S.); constanza.potilinski@gmail.com (M.C.P.); gaodelacalle@gmail.com (G.O.); 2 Departamento de Oftalmología, Hospital Universitario Austral, Pilar, Buenos Aires B1629, Argentina; 3 Consejo Nacional de Investigaciones Cientificas y Técnicas (CONICET), Buenos Aires C1425, Argentina; paulo.maffia@unahur.edu.ar; 4 Centro de Estudios Farmacológicos y Botánicos (CEFYBO), Facultad de Medicina, Consejo Nacional de Investigaciones Científicas y Técnicas, Universidad de Buenos Aires, Buenos Aires C1121, Argentina; guerrieri_diego@hotmail.com (D.G.); echuluyan@fmed.uba.ar (E.C.); 5 Departamento de Microbiología, Facultad de Medicina, Universidad de Buenos Aires, Parasitología e Inmunología, Buenos Aires C1121, Argentina

**Keywords:** SLPI, cementoin, transglutaminase, corneal neovascularization, corneal wound healing, angiogenesis, NF-κB, corneal inflammation, alkali injury

## Abstract

Corneal alkali burns represent one of the most severe forms of ocular surface injury and frequently result in persistent inflammation, corneal neovascularization, stromal remodeling, and permanent visual impairment. Current therapeutic approaches incompletely control the inflammatory mechanisms that sustain pathological angiogenesis and tissue disorganization. In this study, we evaluated the effects of a transglutaminase-binding fusion protein (FP) in a rat model of alkali-induced corneal injury. Following standardized alkali burns, animals were treated topically with FP, secretory leukocyte protease inhibitor (SLPI), or Buffer. Corneal epithelial healing, opacity, and neovascularization were assessed clinically and by digital image-based quantification, while histological and immunofluorescence analyses were used to evaluate stromal organization and vascular invasion. Molecular mechanisms were investigated by RT-qPCR and Western blot analysis of key inflammatory, angiogenic, and signaling mediators. FP treatment significantly accelerated corneal re-epithelialization, reduced corneal opacity, and markedly attenuated corneal neovascularization compared to SLPI and Buffer controls. These effects were associated with coordinated downregulation of pro-inflammatory cytokines and angiogenic mediators, including TNF-α, IL-17, VEGF, and cPLA_2_. Notably, FP suppressed transglutaminase 2 expression and induced early and sustained downregulation of NF-κB pathway components, identifying modulation of an upstream inflammatory pathway central to corneal angiogenesis and stromal remodeling. Collectively, these findings demonstrate that FP effectively limits inflammation-driven corneal neovascularization and tissue remodeling following alkali injury, supporting its potential as a disease-modifying therapeutic strategy for inflammatory ocular surface disorders.

## 1. Introduction

Corneal alkali burns represent one of the most severe forms of ocular surface injury and remain a major cause of corneal morbidity and vision loss worldwide, particularly in working-age populations [1]. These injuries are characterized by rapid penetration of alkaline agents into ocular tissues through lipid saponification, leading to extensive epithelial loss, stromal damage, and a sustained inflammatory response that frequently culminates in corneal neovascularization (CNV), scarring, and permanent visual impairment [2,3,4,5]. Despite advances in acute management, including prompt irrigation and supportive medical therapy, long-term visual outcomes remain suboptimal in a substantial proportion of patients, reflecting the inability of current treatments to adequately control inflammation-driven tissue remodeling and pathological angiogenesis [3,6].

Corneal neovascularization is a critical determinant of poor prognosis following chemical injury, as it disrupts corneal immune privilege and transparency, promotes chronic inflammation, and predisposes to stromal fibrosis and graft failure in patients requiring corneal transplantation [7,8,9]. The angiogenic response after alkali burns is tightly linked to the activation of pro-inflammatory signaling cascades and the sustained expression of cytokines and growth factors such as tumor necrosis factor alpha (TNF-α), interleukin-17 (IL-17), and vascular endothelial growth factor (VEGF) [7,10,11]. In addition, alkali-induced corneal injury is also influenced by oxidative stress-related signaling and extracellular matrix remodeling pathways. In particular, hypoxia-inducible factor-1 (HIF-1) and matrix metalloproteinases (MMPs) have been implicated in the propagation of corneal inflammation, angiogenesis, and stromal degradation, further contributing to loss of corneal transparency and tissue integrity [12,13,14].

Current therapeutic strategies aimed at controlling post-burn inflammation and CNV rely largely on topical corticosteroids and, in selected cases, anti-VEGF agents [7,15,16]. While corticosteroids remain effective in suppressing acute inflammation, their prolonged use is limited by significant adverse effects, including delayed epithelial healing, increased susceptibility to infection, cataract formation, and steroid-induced ocular hypertension or glaucoma [16,17,18]. Anti-VEGF therapies, although effective in reducing neovascular growth, primarily target downstream angiogenic signaling and do not address upstream inflammatory and tissue remodeling processes that sustain CNV, which may explain their limited durability and variable clinical efficacy in corneal disease [9,19].

In this context, increasing attention has been directed toward molecular regulators that integrate inflammation, angiogenesis, and extracellular matrix remodeling [15]. Transglutaminase 2 (TG2) is a multifunctional enzyme widely expressed in inflamed tissues and has emerged as a critical mediator of wound healing, matrix stabilization, and fibrotic responses [20,21,22]. TG2 expression is upregulated under inflammatory conditions and has been shown to interact with and sustain nuclear factor kappa B (NF-κB) signaling, thereby amplifying pro-inflammatory and pro-angiogenic gene expression programs [21,22,23,24]. In ocular tissues, TG2 has been implicated in pathological remodeling processes relevant to corneal injury and neovascularization, positioning it as a potential upstream therapeutic target [20,22]. Transglutaminase expression has been demonstrated in ocular and periocular tissues, including fibroblasts involved in extracellular matrix organization and wound healing responses, supporting a direct role for TG2 in ocular repair mechanisms [25].

Secretory leukocyte protease inhibitor (SLPI) is an endogenous serine protease inhibitor with well-established anti-inflammatory and immunomodulatory properties. Beyond its antiprotease activity, SLPI and its homolog trappin-2 (elafin) have been shown to regulate inflammatory signaling pathways, including inhibition of NF-κB activation, and to modulate epithelial and immune cell responses at mucosal surfaces [26,27,28,29]. However, the therapeutic efficacy of SLPI applied topically to the ocular surface is limited by rapid clearance and insufficient tissue retention, particularly in the setting of active inflammation.

To overcome these limitations, we previously developed a recombinant fusion protein combining SLPI with the N-terminal domain of trappin-2 (cementoin), a natural substrate for TG2 that enables covalent anchoring to TG2-rich extracellular matrices [30,31,32]. This fusion strategy was designed to enhance local retention and biological activity at sites of inflammation and tissue remodeling. In an initial study, this TG2-binding SLPI fusion protein significantly reduced corneal inflammation and neovascularization following alkali injury in a rat model [33]. Building on this rationale, the present study further investigates the therapeutic effects of this fusion protein (FP) on corneal wound healing after alkali burns, with particular emphasis on its impact on corneal neovascularization, stromal remodeling, and the TG2–NF-κB inflammatory axis.

## 2. Results

### 2.1. Corneal Healing

**Figure 1:**
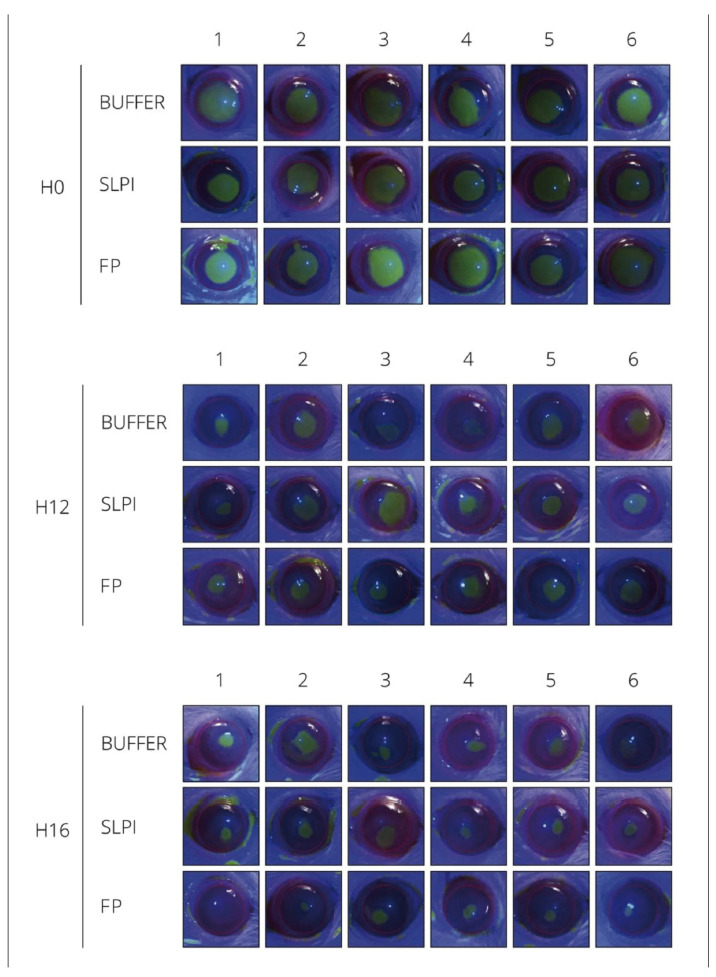
Digital pictures of alkali-injured rat corneas stained with Fluorescein under cobalt blue light at 0, 12 and 16 h. Animals were treated with Buffer, SLPI and FP.

Fluorescein staining performed at 0, 12, and 16 h after alkali injury revealed differences in the rate of corneal epithelial closure among treatment groups. FP-treated corneas exhibited a faster reduction of the epithelial defect over time compared to SLPI- and Buffer-treated eyes. By 16 h post-injury, most FP-treated corneas showed near-complete re-epithelialization, whereas persistent fluorescein-positive areas were still evident in the SLPI and Buffer groups, indicating delayed epithelial repair (Figure 1).

### 2.2. Corneal Opacity

**Figure 2:**
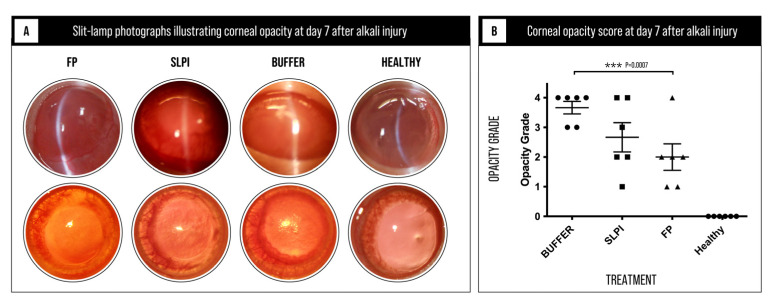
Representative slit-lamp photographs and quantitative assessment of corneal opacity at day 7 after alkali injury. (**A**) Representative slit-lamp photographs of corneas from Buffer-, SLPI-, and FP-treated groups, along with healthy controls. Buffer-treated corneas show marked opacity and loss of transparency, whereas SLPI-treated corneas exhibit moderate opacity. FP-treated corneas demonstrate improved corneal clarity, with preservation of transparency. Healthy corneas show no detectable opacity. (**B**) Quantitative analysis of corneal opacity using a modified Fantes grading scale (0–4). Data are presented as mean ± SEM (*n* = 6 per group). Statistical analysis was performed using the Kruskal–Wallis test followed by Dunn’s multiple comparisons test. Significant differences were observed between groups, with FP-treated corneas showing significantly lower opacity scores compared to Buffer-treated corneas. *** *p* < 0.001.

Corneal opacity was evaluated on day 7 after alkali injury as a clinical indicator of inflammation-associated stromal remodeling and tissue disorganization. Corneal opacity scores differed significantly among the experimental groups (Kruskal–Wallis test, *p* = 0.0007; Figure 2 and Table S2), indicating a differential effect of treatments on corneal transparency.

**Figure 3:**
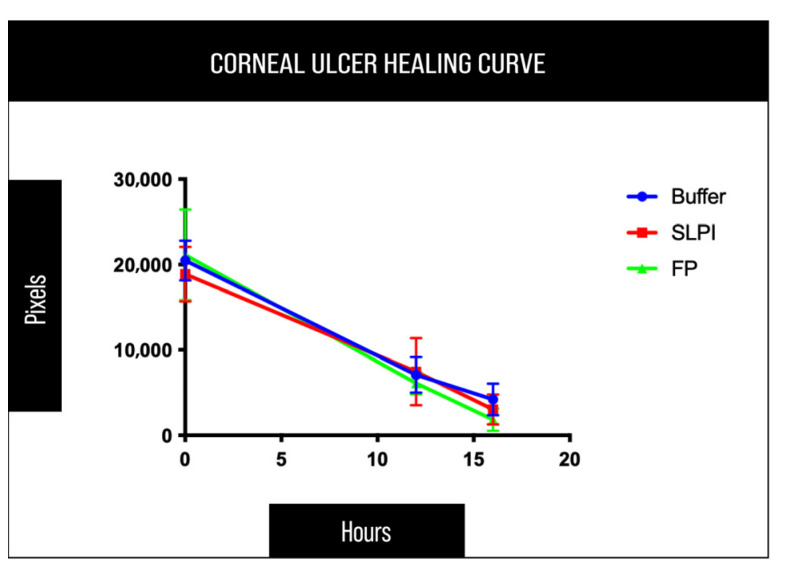
Time-course analysis of corneal epithelial wound closure following alkali injury. Quantification of corneal epithelial defect area assessed by fluorescein staining at 0, 12, and 16 h post-injury. The epithelial defect area was measured in pixels from standardized digital images. FP-treated corneas showed a faster reduction of the fluorescein-stained area over time compared to SLPI- and Buffer-treated corneas.

Quantitative analysis of the epithelial defect area supported these observations. At the final time point (16 h), FP-treated corneas consistently exhibited lower residual epithelial defect values compared to SLPI- and Buffer-treated eyes. However, Kruskal–Wallis test followed by Dunn’s multiple comparisons test did not reveal statistically significant differences among treatment groups (*p* > 0.05), with SLPI-treated corneas showing an intermediate response (Figure 3 and Table S1).

Buffer-treated corneas exhibited a marked loss of transparency, with most eyes reaching high opacity grades, consistent with severe stromal disruption and persistent inflammatory remodeling. SLPI-treated corneas showed a moderate reduction in opacity compared to the Buffer group, although considerable inter-animal variability was observed, with several eyes still displaying moderate to severe haze.

In contrast, FP-treated corneas demonstrated a pronounced reduction in corneal opacity, with significantly lower opacity grades and better preservation of corneal clarity. As expected, healthy control corneas showed no detectable opacity.

Overall, these findings indicate that FP treatment effectively attenuates inflammation-driven stromal remodeling and preserves corneal transparency following alkali injury, supporting its protective effect on corneal structural integrity.

### 2.3. Histological Evaluation of Corneal Remodeling

**Figure 4:**
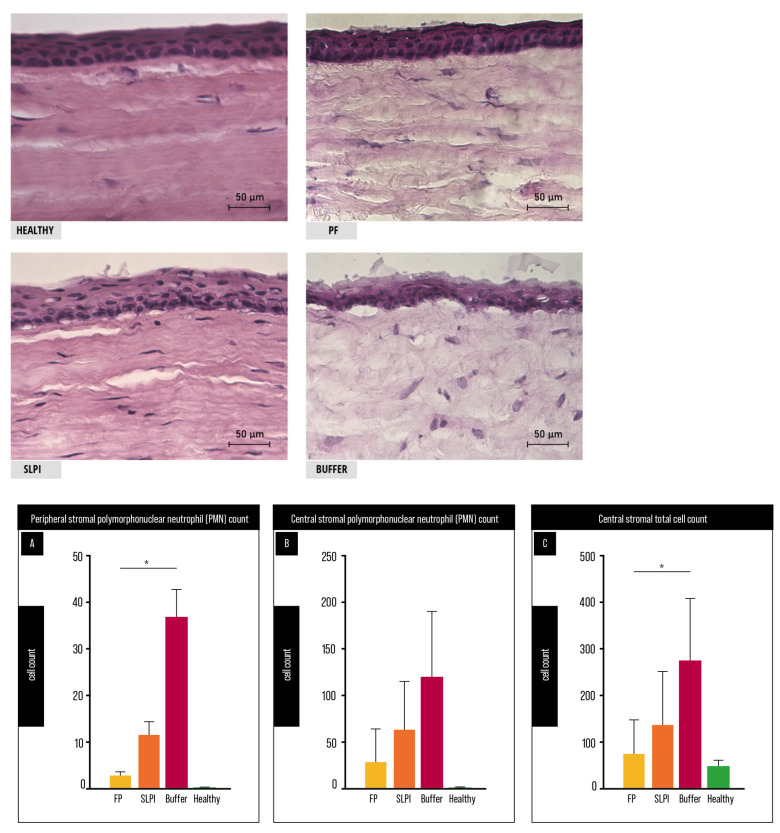
Histological and quantitative assessment of corneal inflammation and stromal remodeling at day 7 after alkali injury. * *p* < 0.05.

Histological examination of corneal sections obtained on day 7 post-injury revealed marked differences in stromal organization and inflammatory cell infiltration among treatment groups (Figure 4). Healthy corneas showed normal lamellar architecture, whereas Buffer-treated corneas exhibited pronounced stromal disorganization, increased cellular infiltration, and loss of normal structure. In contrast, FP-treated corneas displayed improved preservation of stromal architecture with reduced inflammatory infiltration, while SLPI-treated corneas showed an intermediate pattern.

Quantitative analysis supported these observations. Peripheral stromal PMN infiltration (Figure 4B) differed significantly among groups (Friedman test, *p* < 0.0001), with FP-treated corneas showing a significant reduction compared to Buffer-treated controls (Dunn’s test, *p* = 0.0437). No significant difference was observed between SLPI- and Buffer-treated groups. In the central cornea (Figure 4A), PMN counts did not differ significantly between treatment groups.

Total stromal cellularity in the central cornea (Figure 4C) also differed significantly among groups (Friedman test, *p* = 0.0006). Post hoc analysis revealed that FP-treated corneas exhibited significantly reduced cellularity compared to Buffer-treated controls (Dunn’s test, *p* = 0.0405), whereas SLPI-treated corneas did not show a significant difference. FP-treated corneas showed cellularity values closer to those observed in healthy controls, although this difference did not reach statistical significance.

Representative H&E-stained corneal sections show preserved stromal architecture in healthy corneas, marked disorganization and cellular infiltration in Buffer-treated corneas, and improved structural preservation in FP-treated corneas, with SLPI showing an intermediate pattern. (A) Central stromal PMN count. No significant differences between groups. (B) Peripheral stromal PMN count. FP-treated corneas showed reduced PMN infiltration compared to Buffer (* *p* < 0.05). (C) Central stromal total cell count. FP-treated corneas showed reduced cellularity compared to Buffer (* *p* < 0.05). Cells were quantified in central and peripheral stromal fields at 40× magnification (n = 6 per group). Friedman test with Dunn’s multiple comparisons; comparisons vs Buffer. Scale bar: 50 μm.

### 2.4. Corneal Neovascularization

**Figure 5:**
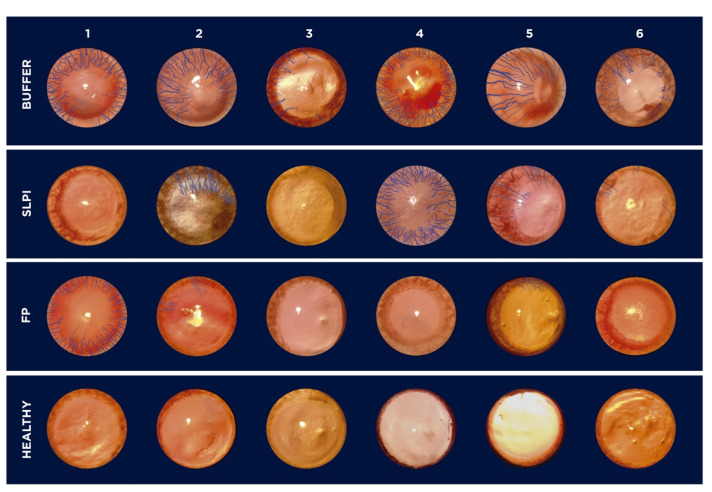
Digital images of alkali-injured rat corneas treated with Buffer, SLPI, FP, and healthy controls at day 7. Each row corresponds to a treatment group: Buffer (**top**), SLPI, FP, and healthy (**bottom**). Columns 1–6 identify individual animals within each group. Corneal neovascularization is marked in blue. All animals in the Buffer group exhibit significant neovascular growth. SLPI-treated corneas show variable response, with some displaying minimal vascularization. FP treatment results in a noticeable reduction in corneal neovascularization compared to Buffer and SLPI. Healthy corneas show no vascular invasion, as expected.

Corneal neovascularization was evaluated at day 7 following alkali injury. Digital images of the ocular surface revealed marked differences among treatment groups (Figure 5). Buffer-treated corneas exhibited extensive neovascularization, with blood vessels extending from the limbus toward the central cornea. In contrast, FP-treated corneas showed a pronounced reduction in neovascular growth, with vessels largely confined to the peripheral cornea. SLPI-treated eyes displayed an intermediate phenotype, with variable degrees of neovascularization among animals.

**Figure 6:**
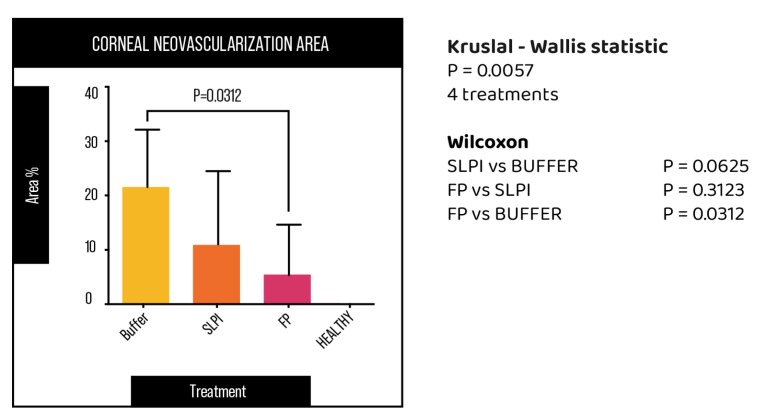
Graphic representing the percentage of corneal area covered by neovascularization in alkali-injured rat corneas treated with Buffer, SLPI, and FP and healthy corneas at day 7.

Quantitative analysis of the neovascularized surface area confirmed these observations (Figure 6). FP-treated corneas exhibited the lowest percentage of vascularized area, which was significantly reduced compared with the Buffer group (*p* = 0.031). Although SLPI treatment showed a trend toward reduced neovascularization compared with Buffer, this difference did not reach statistical significance.

**Figure 7:**
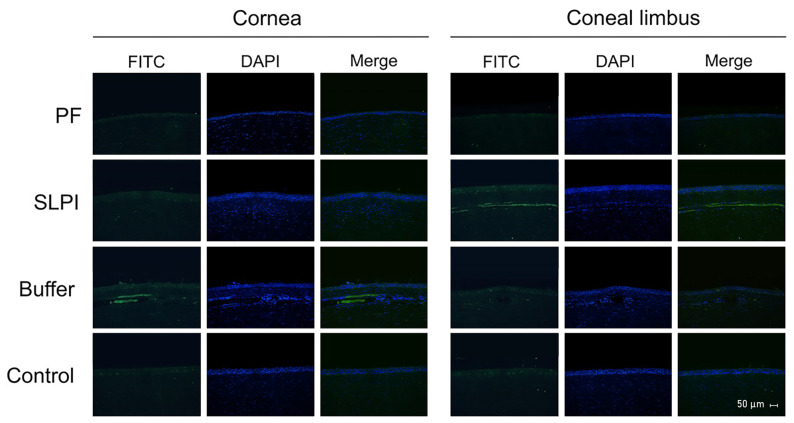
Immunofluorescence analysis of corneal and limbal neovascularization in treated and control groups at day 7 post-alkali injury.Representative cross-sections of the cornea (**left** panels) and limbus (**right** panels) stained with FITC (green, indicating blood vessels) and DAPI (blue, nuclear counterstain). Corneas from Buffer-treated animals show intense neovascularization in both cornea and limbus. SLPI-treated eyes show a moderate vascular signal, reduced compared to Buffer. FP-treated corneas display minimal neovascularization, with weaker FITC signal, particularly at the limbus. Healthy control corneas show no evidence of neovascularization. Images were acquired at 40× magnification. Scale bar = 50 µm.

Immunofluorescence analysis further supported these findings (Figure 7), demonstrating a reduced density of corneal blood vessels in FP-treated eyes, particularly in the limbal and anterior stromal regions. In contrast, Buffer-treated corneas showed dense and disorganized vascular networks extending into the corneal stroma. SLPI-treated corneas exhibited partial attenuation of vascular density.

Histological assessment provided additional insight into the spatial characteristics of neovascular growth (Table 1). In Buffer-treated corneas, neovessels extended across multiple consecutive microscopic fields from the limbus toward the central cornea and penetrated deeper stromal layers. FP treatment markedly limited both the radial extension and stromal penetration of neovessels, whereas SLPI-treated corneas displayed an intermediate pattern. Together, these observations indicate that FP not only reduces the extent of corneal neovascularization but also restricts its invasion into deeper stromal compartments.

### 2.5. Gene Expression Analysis

**Figure 8:**
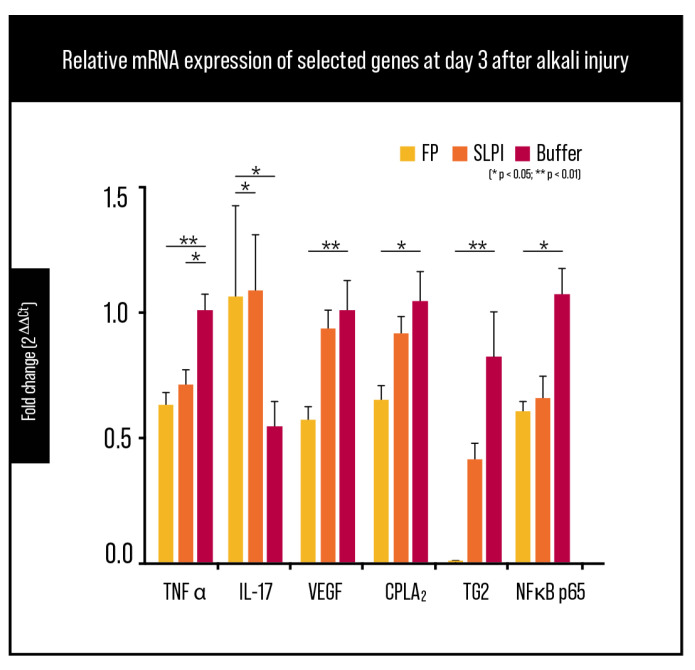
Relative mRNA expression of inflammatory and angiogenic markers at day 3 after alkali injury. Gene expression levels of TNF-α, IL-17A, VEGF, cPLA_2_, RELA, and TG2 were analyzed by RT-qPCR in corneal tissues and normalized to ACTB. Data are presented as [mean ± SEM/median (IQR)] (*n* = 6 per group). Comparisons were performed versus Buffer-treated corneas. * *p* < 0.05; ** *p* < 0.01.

Gene expression analysis by RT-qPCR at day 3 post-alkali injury showed a coordinated modulation of inflammatory, angiogenic, and signaling mediators across treatment groups (Figure 8). TNF-α mRNA levels were reduced in treated corneas compared with Buffer, with a stronger effect in the FP group; SLPI also showed a decrease versus Buffer, consistent with a partial but less pronounced anti-inflammatory response (Figure 8; statistical significance as indicated in the figure).

IL-17A transcripts were significantly lower in FP-treated corneas compared with Buffer (Figure 8). Given the early post-injury time point, this finding is more likely to reflect attenuation of IL-17-associated inflammatory signaling within the acute inflammatory response rather than evidence of an established adaptive Th17 response.

VEGF mRNA, a central driver of corneal neovascularization, exhibited the most pronounced reduction in FP-treated eyes, reaching near-baseline levels comparable to healthy corneas (Figure 8). SLPI also showed a downward shift versus Buffer (Figure 8; significance as indicated), whereas FP consistently showed the lowest VEGF expression among injured groups.

In addition, cPLA_2_ expression (PLA2G4A) showed a moderate downward trend in FP-treated corneas compared with Buffer controls (Figure 8), suggesting broader dampening of pro-inflammatory lipid mediator pathways.

At the transcriptional level, RELA (NF-κB p65) mRNA showed a reduction in FP-treated corneas, although values remained in a similar range to SLPI in this dataset (Figure 8). Finally, TG2 mRNA expression was significantly reduced in FP-treated corneas compared to both Buffer and SLPI groups (Figure 8), indicating effective inhibition of TG2 upregulation associated with injury-driven stromal remodeling.

Overall, these results support that FP shifts the early post-injury corneal transcriptional program toward reduced inflammatory signaling, diminished angiogenic drive, and lower TG2 induction.

### 2.6. Protein Expression of Inflammatory, Angiogenic, and Signaling Mediators

**Figure 9:**
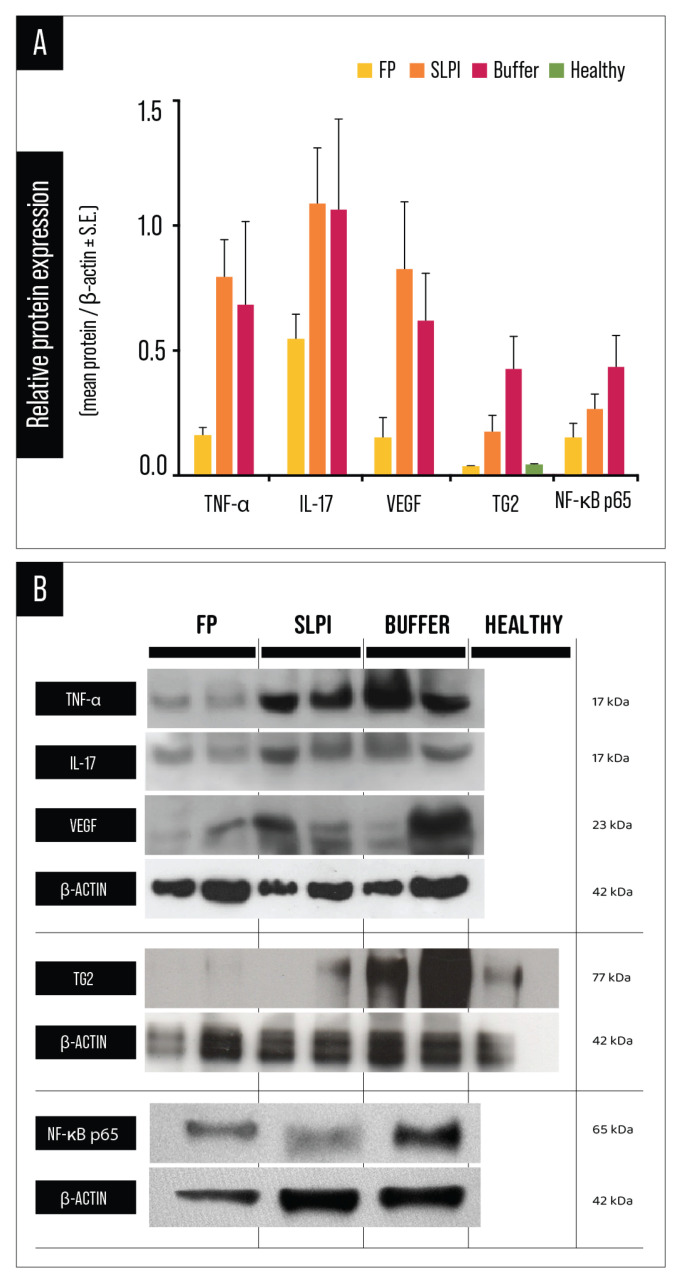
Protein expression analysis of inflammatory and angiogenic markers in corneal tissue following alkali injury. (**A**) Densitometric quantification of TNF-α, IL-17, VEGF, TG2, and NF-κB p65 protein levels in FP-, SLPI-, Buffer-treated, and healthy control corneas. Protein expression was normalized to β-actin and expressed as mean ± SEM. (**B**) Representative Western blot images corresponding to each analyzed protein, including β-actin as loading control. Molecular weights are indicated on the right.

Western blot analysis followed by densitometric quantification demonstrated a consistent modulation of inflammatory, angiogenic, and signaling mediators at the protein level across treatment groups (Figure 9).

TNF-α protein levels were significantly reduced in FP-treated corneas compared to both SLPI and Buffer groups, as determined by one-way ANOVA followed by Tukey’s multiple comparisons test (*p* < 0.01; Figure 9A and Table S3), indicating a robust attenuation of injury-induced inflammatory signaling. SLPI treatment also resulted in a partial reduction compared to Buffer, although to a lesser extent than FP.

IL-17 protein expression was significantly reduced in FP-treated corneas compared to both SLPI and Buffer groups, as determined by one-way ANOVA followed by Tukey’s multiple comparisons test (*p* < 0.01; Figure 9B and Table S4). No significant difference was observed between SLPI and Buffer groups. These findings indicate that FP treatment effectively suppresses IL-17 upregulation following alkali injury.

VEGF protein expression, a key mediator of corneal neovascularization, was markedly reduced in FP-treated corneas compared to both SLPI (*p* < 0.0001) and Buffer groups (*p* = 0.0015). SLPI treatment also showed a trend toward reduction relative to Buffer, although this difference did not reach statistical significance. These results confirm that FP treatment achieved the most pronounced suppression of VEGF expression (Figure 9; Table S5).

TG2 protein expression was significantly downregulated in FP-treated corneas compared to both Buffer (*p* < 0.0001) and SLPI groups (*p* = 0.0459). Moreover, SLPI treatment also significantly reduced TG2 levels relative to Buffer (*p* = 0.0008), indicating that both treatments modulate TG2-associated remodeling processes, with FP exerting the strongest inhibitory effect (Figure 9; Table S6).

NF-κB p65 protein expression was significantly modulated by treatment. One-way ANOVA revealed significant differences among experimental groups, and Tukey’s multiple comparisons test showed that FP-treated corneas exhibited markedly lower NF-κB p65 levels compared to both Buffer (adjusted *p* < 0.0001) and SLPI groups (adjusted *p* = 0.0112). In addition, SLPI treatment significantly reduced NF-κB p65 expression relative to Buffer (adjusted *p* = 0.0041), although the magnitude of reduction was greater in the FP group. These results indicate that FP more effectively attenuates NF-κB signaling at the protein level, consistent with its broader anti-inflammatory and anti-angiogenic effects (Figure 9 and Table S7).

Collectively, these results demonstrate that FP exerts a broad and coordinated inhibitory effect on key inflammatory, angiogenic, and signaling mediators at the protein level, reinforcing its capacity to limit inflammation-driven neovascularization and stromal remodeling following alkali injury.

### 2.7. Modulation of NF-κB Pathway

**Figure 10:**
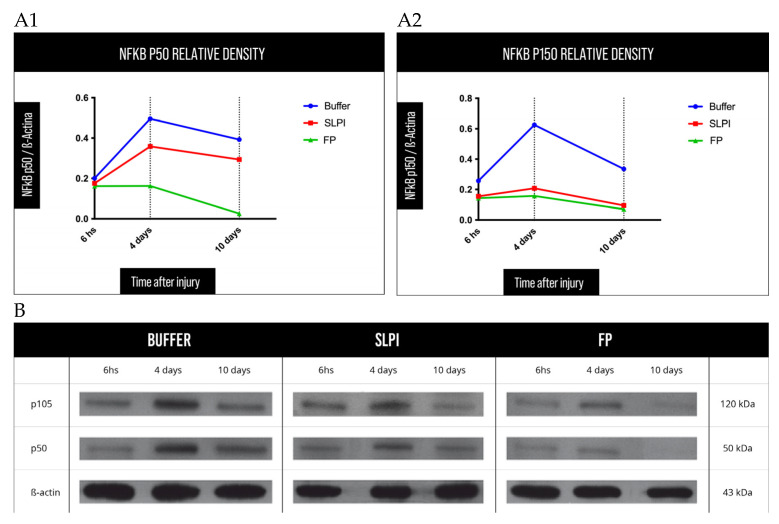
Western blot analysis of NF-κB pathway modulation in rat corneas after alkali injury. (**A1**,**A2**) Graphs of NF-κB p50 and NF-κB p105 relative density, compared with β-actin, measured at 6 h, at 4 days, and at 10 days after alkali injury. Quantitative densitometric analysis of NF-κB subunits p105 and p50, normalized to β-actin, at three time points post-injury: 6 h, 4 days, and 10 days. The graphs show a consistent downregulation of both NF-κB subunits in FP-treated corneas, especially evident at early time points (6 h and 4 d), indicating a time-dependent inhibition of the inflammatory cascade. SLPI shows a moderate reduction, while Buffer-treated groups maintain higher expression levels across all time points. (**B**) Western blot of NF-κB p105, p50 and β-actin at the three times measured after alkali injury of rat corneas treated with Buffer, SLPI and FP.

To further characterize the anti-inflammatory mechanisms of FP, temporal changes in NF-κB protein expression were evaluated at early and intermediate time points following alkali injury (Figure 10). NF-κB pathway protein expression was significantly attenuated in FP-treated corneas compared to the Buffer group at all evaluated time points, indicating effective suppression of NF-κB-mediated inflammatory signaling.

Densitometric analysis revealed a distinct temporal profile of NF-κB expression among treatment groups. In Buffer-treated corneas, NF-κB p50 and p105 levels showed an early peak at 6 h post-injury and remained elevated at days 4 and 10, consistent with sustained inflammatory activation. In contrast, FP treatment induced a marked reduction in both NF-κB subunits as early as 6 h post-injury, with this inhibitory effect maintained throughout the observation period.

SLPI-treated corneas displayed an intermediate expression pattern, characterized by partial suppression of NF-κB p50 and p105 at all time points, but without the early and sustained inhibition observed in the FP group.

Overall, these results demonstrate that FP induces a rapid and durable attenuation of NF-κB signaling following alkali injury, providing mechanistic support for its broad anti-inflammatory and anti-angiogenic effects observed at the molecular, histological, and clinical levels.

## 3. Discussion

Corneal alkali burns remain a major cause of severe ocular surface damage, frequently leading to chronic inflammation, corneal neovascularization (CNV), stromal remodeling, and permanent visual impairment despite advances in acute clinical management [1,2,3,4,5]. The persistence of these adverse outcomes highlights the need for therapeutic strategies capable of modulating not only early inflammatory events but also the downstream molecular pathways that drive pathological angiogenesis and fibrotic remodeling [6,7,10].

In the present study, we demonstrate that treatment with FP significantly improves corneal healing outcomes following alkali injury, as evidenced by accelerated epithelial closure, reduced corneal opacity, marked inhibition of CNV, and coordinated suppression of inflammatory, angiogenic, and signaling mediators at both transcriptional and protein levels. Overall, FP showed the most consistent therapeutic profile among the tested treatments. However, differences between FP and SLPI were not statistically significant for all evaluated parameters, and therefore comparative interpretation should be made cautiously.

Rapid restoration of epithelial integrity represents a critical determinant of corneal recovery after chemical injury, as persistent epithelial defects perpetuate inflammation and stromal damage [2,4]. FP-treated corneas exhibited significantly faster re-epithelialization compared with Buffer and SLPI groups, suggesting that early control of inflammation facilitates epithelial repair. This accelerated wound closure likely contributes to the downstream reduction in stromal remodeling and neovascularization observed at later time points.

Corneal opacity was assessed as a clinically relevant surrogate marker of stromal remodeling rather than acute injury severity. Given the high reproducibility of the alkali burn model used and the preservation of the sclerocorneal limbus, differences in opacity primarily reflect inflammation-driven alterations in stromal organization, extracellular matrix integrity, and cellular infiltration [6,15]. FP treatment resulted in a pronounced preservation of corneal transparency, consistent with histological findings showing improved stromal organization and reduced inflammatory infiltration. These observations underscore the ability of FP to modulate tissue remodeling processes that directly impact visual outcomes.

CNV is a central pathological feature following corneal chemical injury, contributing to loss of immune privilege, chronic inflammation, and poor prognosis in patients requiring corneal transplantation [7,8,10,34]. FP markedly reduced both the surface area and stromal penetration of neovessels, as demonstrated by digital image-based quantification and histological assessment. Importantly, histological analysis revealed that FP limited vascular ingrowth predominantly to superficial stromal layers, whereas Buffer-treated corneas showed deeper and more extensive vascular penetration. This distinction is clinically relevant, as deeper stromal neovascularization is associated with more aggressive angiogenic responses and greater disruption of corneal transparency [15,34].

At the molecular level, FP induced a coordinated downregulation of key inflammatory and angiogenic mediators. RT-qPCR analysis at day 3 post-injury revealed significant reductions in TNF-α, VEGF, and TG2 mRNA expression in FP-treated corneas, with more modest or variable effects observed with SLPI. While IL-17 transcripts were also reduced in FP-treated corneas, this finding should be interpreted in the context of early sterile injury, where IL-17 may derive from innate immune sources such as γδ T cells, innate lymphoid cells, or neutrophils rather than classical Th17 cells [11,29]. Nevertheless, the overall transcriptional profile indicates an early shift away from pro-inflammatory and pro-angiogenic signaling in FP-treated eyes.

Protein expression analyses further supported these findings. FP significantly reduced TNF-α, IL-17, VEGF, and TG2 protein levels compared with Buffer-treated corneas, with SLPI again showing intermediate effects. The reduction in TG2 expression is particularly relevant given its established role as a multifunctional regulator of wound healing, extracellular matrix stabilization, and fibrotic responses [20,23,35,36]. TG2 has also been shown to sustain NF-κB activation through positive feedback mechanisms, thereby amplifying inflammatory and angiogenic signaling [21,37,38]. Thus, inhibition of TG2 by FP may represent a key upstream event contributing to the broader suppression of inflammatory pathways observed in this study.

Consistent with this hypothesis, FP treatment resulted in significant modulation of the NF-κB signaling axis at both transcriptional and protein levels. Beyond static measurements, chronological analysis revealed a temporal decrease in NF-κB p105 and p50 protein expression at day 4, followed by a reduction in total p65 at day 7, observed predominantly in FP-treated animals. This staged downregulation of the canonical NF-κB pathway is compatible with the activation of an active resolution phase in a model of corneal injury, rather than nonspecific pathway suppression [37,39,40]. Such coordinated modulation supports the notion that FP prevents the establishment of sustained NF-κB activation, thereby limiting progression toward chronic inflammatory states, including Th17-type inflammatory programs, which have been implicated in persistent corneal inflammation and pathological neovascularization [7,11].

Current therapeutic approaches for CNV, including corticosteroids and anti-VEGF agents, primarily target downstream effectors and are limited by significant side effects or incomplete efficacy [6,16,17]. In contrast, FP appears to act upstream by simultaneously modulating inflammatory signaling, angiogenic drivers, and extracellular matrix remodeling. This multi-level mechanism may explain its superior efficacy compared with SLPI alone and highlights the advantage of anchoring anti-inflammatory molecules within TG2-rich inflamed tissues to enhance local retention and biological impact.

The present study builds upon previous work demonstrating the efficacy of TG2-binding fusion proteins in ocular inflammation [30,33] and extends these findings by providing mechanistic insight into the TG2–NF-κB axis in corneal alkali injury.

The lack of a healthy control across molecular analyses represents a limitation of this study. The Animal Ethics Committee recommended minimizing animal use and considered the Buffer-treated injured group an appropriate reference condition to evaluate treatment effects within the same injury context.

To address this limitation and provide a physiological reference, a healthy control group was included in the Western blot analysis of TG2, allowing contextualization of injury-induced upregulation and treatment-related modulation. However, healthy controls were not included in the full panel of molecular analyses (RT-qPCR and additional protein targets), and therefore comparisons are primarily restricted to treatment effects within the injured model.

An additional limitation of the study is the small sample size used for the kinetic Western blot analysis at 6 h, 4 d, and 10 d post-injury. These experiments were intended as an exploratory temporal assessment of NF-κB pathway modulation and should therefore be interpreted as supportive mechanistic data rather than definitive quantitative evidence in isolation.

While further studies are warranted to explore long-term outcomes, expand molecular validation, and strengthen translational applicability, our results support FP as a promising therapeutic strategy. Specifically, FP demonstrated consistent effects in reducing inflammation-driven corneal neovascularization and promoting stromal remodeling following chemical injury.

## 4. Materials and Methods

### 4.1. Animals

A total of 81 male Sprague–Dawley rats (250–300 g, approximately 8 weeks of age) were used in this study. Animals were obtained from the Comisión Nacional de Energía Atómica (CNEA, Buenos Aires, Argentina) and housed under standard laboratory conditions with a 12 h light/dark cycle, controlled temperature, and ad libitum access to food and water.

All experimental procedures were conducted in accordance with the ARVO Statement for the Use of Animals in Ophthalmic and Vision Research and were approved by the Institutional Animal Care and Use Committee of Austral University (CICUAL).

Animals were randomly assigned to treatment groups, and all experimental procedures, treatments, and outcome assessments were performed in a masked fashion. Each animal contributed only one eye to the study: the right eye was subjected to alkali injury, while the left eye was left untreated and served as an internal healthy control. No animals were excluded from the analysis, and no procedure-related mortality occurred. The allocation of animals to the different experimental cohorts and analytical endpoints is summarized in Table 2.

Rats subjected to corneal alkali injury were allocated to four experimental cohorts depending on the analyses performed. Cohort 1 (*n* = 18) was used for clinical imaging, histology, and immunofluorescence analyses. Corneal epithelial wound closure was assessed by fluorescein staining at 0, 12, and 16 h, while corneal opacity measurement, corneal neovascularization (CNV) area measurement, histological evaluation (H&E), and immunofluorescence staining (PECAM-1/CD31) were performed at day 7 post-injury. Cohort 2 (*n* = 27) was used to evaluate NF-κB pathway kinetics by Western blot at 6 h, 4 days, and 10 days after injury. Cohort 3 (*n* = 18) was used for Western blot analysis of inflammatory and angiogenic mediators (TNF-α, IL-17, VEGF, TG2, and NF-κB p65) at day 7. Cohort 4 (*n* = 18) was used for gene expression analysis by RT-qPCR at day 3 post-injury, assessing TNF-α, IL-17A, VEGF, cPLA_2_, RELA, and TG2. In total, 81 animals were included in the study.

### 4.2. Induction of Corneal Alkali Injury

Corneal alkali injury was induced using a standardized and reproducible rat model of inflammation-induced corneal neovascularization, as previously described, with minor modifications [15,41]. Animals were anesthetized with inhaled isoflurane, and topical anesthesia was achieved by instillation of proparacaine hydrochloride 0.5% eye drops (Poen-caína^®^, Poen, Argentina).

A circular filter paper disk (3.0 mm diameter) soaked in 1 mol/L sodium hydroxide (NaOH) solution was placed on the central corneal surface of the right eye for 40 s under direct visualization. After removal of the disk, the ocular surface was immediately irrigated with 5 mL of sterile 0.9% saline solution using a 25 G cannula to ensure complete removal of residual alkali.

This model produces a consistent epithelial and anterior stromal injury with preservation of the sclerocorneal limbus and has been widely used to study inflammatory signaling, corneal neovascularization, and therapeutic interventions targeting these processes [15,39,40,41].

### 4.3. Study Design and Treatment Groups

Following alkali injury, animals were allocated into three experimental groups (*n* = 6 per group at each experimental time point): (A) Buffer-treated group, receiving vehicle solution, (B) SLPI-treated group and (C) FP-treated group.

Treatments were administered topically to the injured right eye according to the experimental protocol. Group allocation was coded (A, B, C) to ensure masking of investigators throughout treatment administration, data acquisition, and analysis. Animals from different groups were housed separately, and treatments were never mixed between groups.

For corneal epithelial wound closure, corneal neovascularization assessment at day 7, histological analysis, and immunofluorescence studies, the same animal cohort (*n* = 18 total) was used. For tissue-based analyses, eyes were collected at the corresponding endpoint, and serial corneal sections were prepared from each sample. Separate sections were then allocated to H&E staining and immunofluorescence analysis.

For kinetic Western blot analysis of the NF-κB pathway, separate biological cohorts were studied at 6 h, 4 days, and 10 days post-injury (*n* = 9 animals per time point; 3 animals per treatment group). An additional independent cohort of 18 animals (*n* = 6 per group) was used for Western blot analysis at day 7 post-injury.

For gene expression analysis by RT-qPCR, one cohort of 18 animals (*n* = 6 per group) was used to evaluate mRNA levels of TNF-α, IL-17, VEGF, cPLA_2_, NF-κB p65, and TG2.

At the end of each experimental time point, animals were euthanized using a CO_2_ chamber in accordance with institutional guidelines.

### 4.4. FP Purification

The recombinant fusion protein (FP) was produced using a bacterial expression system based on Escherichia coli BL21-CodonPlus-RIL cells (Stratagene, Darmstadt, Germany), as previously described [27]. Following induction of protein expression, bacterial cultures were harvested, and cell pellets were lysed by ultrasonic disruption. The lysates were centrifuged to remove insoluble debris, and the clarified supernatant containing the soluble recombinant protein was collected.

Purification of FP was performed by immobilized metal affinity chromatography using a nickel–nitrilotriacetic acid (Ni-NTA) resin (Qiagen GmbH, Hilden, Germany), exploiting the histidine tag engineered into the fusion protein. After binding, the column was washed to remove non-specifically bound proteins, and FP was eluted using an imidazole-containing buffer (50 mM NaH_2_PO_4_, 300 mM NaCl, 250 mM imidazole, pH 8.0).

To reduce residual imidazole, eluted protein fractions were dialyzed overnight at 4 °C against phosphate buffer, achieving a final imidazole concentration of 2.5 mM. The dialyzed protein was then recovered, aliquoted, and stored at −80 °C. The corresponding dialysis buffer was retained and used as vehicle control in subsequent experiments.

To minimize contamination with bacterial endotoxins, FP preparations were treated with polymyxin B–agarose resin, followed by centrifugation to recover the endotoxin-depleted protein. Protein concentration was determined using the MicroBCA protein assay (Pierce, Rockford, IL, USA), according to the manufacturer’s instructions.

### 4.5. Clinical Assessments

#### 4.5.1. Assessment of Corneal Wound Closure Time

Corneal epithelial wound healing following alkali-induced injury was evaluated by fluorescein staining. Areas of persistent epithelial defect retained fluorescein, whereas absence of staining indicated complete re-epithelialization.

Six eyes per treatment group were examined at regular 6 h intervals after injury. Fluorescein was instilled, and corneas were evaluated under an operating microscope (Zeiss S4, Göttingen, Germany) equipped with cobalt blue illumination. High-resolution digital images were acquired at 0, 12, and 16 h post-injury using a digital camera (Nikon Coolpix S3500, 20.1 megapixels, Tokyo, Japan) coupled to the microscope via an ocular adapter.

Digital images were processed using Adobe Photoshop (Adobe Systems, San Jose, CA, USA). To allow quantitative comparison, corneal dimensions were normalized across images. The ulcerated area was delineated and quantified in pixels, and epithelial defect size was expressed as a percentage of the total corneal area. Data were reported as the mean ± standard error of the mean (SEM). Statistical comparisons among groups were performed using the Kruskal–Wallis test.

#### 4.5.2. Corneal Neovascularization Assessment

To assess the extent of corneal neovascularization (CNV), digital images of alkali-injured rat corneas were captured on day 7 post-injury for each treatment group (Buffer, SLPI, and FP). Topical tropicamide (without phenylephrine) was instilled (10 μL) 30 min before image acquisition to enhance visualization of the red fundus reflex. Animals were sedated using inhaled isoflurane.

For each eye, five digital photographs were obtained using an operating microscope (Zeiss S4, Göttingen, Germany) coupled to a digital camera (Nikon Coolpix S3500, 20.1 megapixels, Tokyo, Japan) fitted with a microscope ocular adaptor. Images included one frontal corneal view and four oblique views corresponding to each ocular quadrant. All images were acquired under identical magnification, illumination, and focus settings.

To ensure accurate and reproducible comparisons, all corneal images were standardized by aligning the limbal borders to a uniform diameter across samples. Image processing and quantitative analysis were performed using Adobe Photoshop (Adobe Systems, San Jose, CA, USA). A master file with predefined image size and resolution was created and used as a reference template. Within this file, a circular region of interest (ROI) of fixed diameter was drawn to represent the corneal surface.

Individual corneal images were imported as separate layers beneath the circular ROI and manually aligned so that the sclerocorneal limbus precisely coincided with the predefined circular outline. After alignment, the content within the circular ROI was cropped, ensuring that all analyzed corneal images shared identical size, resolution, and total pixel count.

Corneal blood vessels were manually delineated using the pencil tool under high magnification (zoom) and highlighted in blue. In areas where corneal vessels overlapped with iris vessels, particularly in the extreme peripheral cornea, quadrant images were reviewed to confirm vessel localization and direction, ensuring accurate identification of corneal neovascularization. The blue-colored vascular area was selected, and the corresponding pixel count was obtained from the Photoshop information panel. In parallel, the total pixel area of the circular ROI was measured for each cornea.

Corneal neovascularization was expressed as the percentage of vascularized area relative to total corneal area. A representative example of the digital image-based workflow used for CNV quantification is shown in Figure S1. Group comparisons were performed using the Kruskal–Wallis test.

#### 4.5.3. Corneal Opacity Assessment

Corneal opacity was evaluated as an indirect indicator of stromal remodeling and tissue disorganization secondary to inflammation, rather than as a surrogate marker of initial injury severity. Given the high reproducibility of the alkali injury model employed in this study—characterized by a standardized epithelial and anterior stromal insult, preservation of the sclerocorneal limbus, and minimal inter-animal variability in lesion extent—a specific alkali burn severity classification was not applied. Instead, corneal opacity assessment was used to capture differences in the inflammatory response and subsequent stromal reorganization among treatment groups. This approach allows the evaluation of transparency loss associated with extracellular matrix disruption, inflammatory cell infiltration, and alterations in collagen architecture, which are key determinants of corneal clarity and visual outcome.

Corneal clarity and haze were examined on day 7 post-injury using slit-lamp biomicroscopy (Haag-Streit, Köniz, Switzerland). Opacity was graded according to a modified version of the Fantes et al. classification system [42], as follows: Grade 0: completely transparent cornea; Grade 0.5: minimal haze detectable only under oblique illumination; Grade 1: mild haze without obscuration of iris details; Grade 2: moderate haze with partial visibility of iris structures; Grade 3: marked opacity readily visible under direct illumination with significant obscuration of iris details; and Grade 4: severe opacity with no visible anterior chamber structures. Representative slit-lamp images illustrating the assessment of corneal opacity are shown in Figure S2. Statistical comparisons among treatment groups were performed using the Kruskal–Wallis test.

### 4.6. Histological and Immunofluorescence Analysis of Corneal Tissues

To investigate structural and cellular responses following alkali-induced corneal injury, corneal tissues were collected at days 3 and 7 post-injury for histological and immunofluorescence analyses. Animals were euthanized by CO_2_ inhalation, eyeballs were enucleated, and corneas were carefully dissected under a stereomicroscope. Tissues were fixed in 10% neutral buffered formalin, cryoprotected, embedded in optimal cutting temperature (OCT) compound, and stored at low temperature until sectioning. Serial cryosections of 8 µm thickness were obtained from each corneal sample and mounted on separate slides. Sections were allocated differentially for H&E staining or CD31 immunofluorescence analysis, so that each slide underwent only one staining protocol.

For histological evaluation, sections were stained with hematoxylin and eosin (H&E) to assess epithelial integrity and stratification, stromal cellular infiltration, extracellular matrix disorganization and remodeling, and the presence of neovascular structures. For quantitative analysis, inflammatory cell infiltration and total stromal cellularity were evaluated in both central and peripheral corneal regions. Polymorphonuclear neutrophils (PMNs) were identified based on characteristic nuclear morphology (multilobed nuclei) and counted manually. Total stromal cell counts included all nucleated cells within the stromal compartment, regardless of cell type. Quantification was performed using light microscopy at 40× magnification. For each cornea, one representative central stromal field and one peripheral stromal field (adjacent to the limbal region) were analyzed. Cell counts were performed in a masked fashion to avoid observer bias. Results were expressed as the number of cells per field.

For immunofluorescence analysis, endothelial cells were identified using anti-CD31 antibody (ab7388, Abcam, Cambridge, MA, USA) immunostaining, while cell nuclei were counterstained with DAPI. Fluorescent signals were detected using appropriate filter settings, and images were acquired using identical exposure parameters across experimental groups.

Neovessels were classified according to their depth of stromal penetration as superficial (anterior third), middle (middle third), or deep (posterior third) stroma. The extent of vascular ingrowth was assessed by counting vessel-containing microscopic fields at 40× magnification across consecutive corneal sections, from the limbal region toward the central cornea. Histological findings regarding neovascular extension and depth were analyzed descriptively and summarized for each experimental group.

### 4.7. RT-qPCR Analysis

Total RNA was isolated from corneal tissues collected at day 3 post-alkali injury using TRIzol reagent (Invitrogen, Carlsbad, CA, USA), according to the manufacturer’s instructions. RNA concentration and purity were assessed by spectrophotometry (NanoDrop, Thermo Fisher, Waltham, MA, USA). One microgram of total RNA from each sample was reverse-transcribed using 200 U of SuperScript II Reverse Transcriptase (Invitrogen, Carlsbad, CA, USA) and 500 ng of oligo (dT) primers, following the manufacturer’s protocol.

Quantitative real-time PCR (RT-qPCR) was performed using gene-specific primers listed in Table 3. Amplification reactions were carried out using SYBR Green PCR Master Mix (Applied Biosystems, Carlsbad, CA, USA) on an Mx3005P real-time PCR system (Stratagene, La Jolla, CA, USA). The thermal cycling protocol consisted of an initial denaturation step at 95 °C for 10 min, followed by 40 amplification cycles of 95 °C for 30 s, 60 °C for 1 min, and 72 °C for 30 s. At the end of each run, a melting curve analysis was performed by increasing the temperature from 60 °C to 95 °C at a rate of 2 °C/min, with fluorescence acquisition every 15 s, to confirm amplification specificity.

Gene expression levels of TNF-α, IL-17, VEGF, cPLA_2_, NF-κB p65, and TG2 were normalized to β-actin (ACTB) as the endogenous reference gene. Relative mRNA expression was calculated using the comparative ΔΔCt method, as previously described by Livak and Schmittgen [43]. Expression values were expressed relative to the Buffer-treated group, which was set to 1, in order to specifically assess the effect of each treatment on injury-induced gene expression. Non-template controls were included in all runs to exclude contamination. All reactions were performed in triplicate for each sample.

### 4.8. Western Blot Analysis

Corneal tissues were collected at the indicated time points after alkali injury and homogenized in ice-cold RIPA lysis buffer supplemented with protease and phosphatase inhibitor cocktails. Tissue lysates were clarified by centrifugation, and total protein concentration was determined using the Bradford assay to ensure equal protein loading across samples.

Equal amounts of protein (20–30 µg per lane) were separated by SDS–PAGE under reducing conditions and transferred onto polyvinylidene difluoride (PVDF) membranes. Membranes were blocked in 5% non-fat dry milk prepared in Tris-buffered saline containing 0.1% Tween-20 (TBST) and subsequently incubated overnight at 4 °C with the following primary antibodies: NF-κB p65 (rabbit, 8242S, Cell Signaling Technology, Danvers, MA, USA), VEGF-A (mouse, ab105219, Abcam, Waltham, MA, USA), TNF-α (rabbit, ab205587, Abcam, Waltham, MA, USA), IL-17 (goat, sc-6076, Santa Cruz Biotechnology, Dallas, TX, USA), TG2 (rabbit, 3557S, Cell Signaling Technology, Danvers, MA, USA), and β-actin (mouse, sc-8432, Santa Cruz Biotechnology, Dallas, TX, USA) used as a loading control.

After washing, membranes were incubated with the appropriate horseradish peroxidase-conjugated secondary antibodies. Immunoreactive bands were detected using an enhanced chemiluminescence (ECL) system and captured with a digital imaging system. For selected experiments, PVDF membranes were subjected to stripping and reprobing procedures to allow sequential detection of multiple target proteins on the same membrane. In these cases, membranes were stripped using a commercially available stripping buffer, re-blocked, and reprobed with the corresponding primary antibodies. β-actin loading controls correspond to the same membrane used for the detection of the respective target proteins.

Band intensities were quantified by densitometric analysis using ImageJ software (version 1, National Institutes of Health, Bethesda, MD, USA). Protein expression levels were normalized to β-actin and expressed as relative values compared to the Buffer-treated group. Statistical analysis of densitometric data was performed using one-way analysis of variance (ANOVA) followed by Tukey’s multiple comparisons test, as detailed in the Statistical Analysis subsection.

### 4.9. Statistical Analysis

Data were analyzed using parametric or non-parametric statistical methods, as appropriate. Normality was assessed using Shapiro–Wilk test. For datasets not assuming normality, comparisons among experimental groups were performed using the Kruskal–Wallis test. For normally distributed data, one-way analysis of variance (ANOVA) followed by Tukey’s multiple comparisons test was used. Results are expressed as mean ± standard error of the mean (SEM), unless otherwise stated. A *p* value < 0.05 was considered statistically significant. Statistical analyses were performed using GraphPad Prism software 10.6.1 (GraphPad Software, San Diego, CA, USA).

Histological assessment of corneal neovascularization extension and depth was analyzed descriptively due to the ordinal and semi-quantitative nature of these measurements.

## 5. Conclusions

The present study demonstrates that the fusion protein Cementoin–SLPI exerts a potent protective effect in an experimental model of alkali-induced corneal injury. FP treatment significantly reduced corneal neovascularization, attenuated stromal remodeling and opacity, and accelerated epithelial recovery. At the molecular level, FP downregulated key inflammatory and angiogenic mediators, including TNF-α, IL-17, VEGF, TG2, and NF-κB p65, indicating a coordinated modulation of inflammation-driven corneal pathology.

Although SLPI alone provided partial protection, the fusion construct consistently produced stronger anti-inflammatory and anti-angiogenic effects, supporting the rationale for targeted tissue delivery strategies.

Collectively, these findings position Cementoin–SLPI as a promising therapeutic candidate for the management of corneal neovascularization and inflammation-associated corneal disorders.

## Figures and Tables

**Table 1:** Extension and depth of corneal neovascularization assessed by histology. Histological assessment of corneal neovascularization evaluating both lateral extension from the limbus toward the central cornea and stromal depth of vascular ingrowth across treatment groups.

**A. Extension of corneal neovascularization.**						
**Treatment Group**	**Field 1**	**Field 2**	**Field 3**	**Field 4**	**Field 5**	**Field 6**
Buffer	6	6	4	3	2	1
SLPI	4	4	3	1	0	0
FP	3	3	1	0	0	0
Healthy	0	0	0	0	0	0
**B. Depth of corneal neovascularization.**						
**Treatment Group**	**Superficial Third**		**Middle Third**		**Deep Third**	
Buffer	6		6		3	
SLPI	4		3		1	
FP	3		0		0	
Healthy	0		0		0	

Extension of corneal neovascularization expressed as the number of 40× histological fields containing blood vessels, evaluated from the limbal region toward the central cornea for each treatment group. Depth of corneal neovascularization classified according to stromal penetration as superficial (anterior third), middle (middle third), or deep (posterior third) stroma. Values represent the number of eyes exhibiting neovessels at each stromal level per treatment group.

**Table 2:** Experimental allocation of animals and analytical endpoints in the rat corneal alkali burn model.

Cohort	Technique	Variables Analyzed	Time Point	Animals
Cohort 1 Clinical and morphological analysis	Clinical imaging	epithelial wound closure	0, 12, 16 h	18
	Clinical imaging	corneal opacity	day 7	
	Clinical imaging	CNV area	day 7	
	Histology (H&E)	inflammatory infiltration, stromal organization, CNV depth	day 7	
	Immunofluorescence	PECAM/CD31 vascular staining	day 7	
Cohort 2 Western blot (NF-κB kinetics)	Western blot	NF-κB p105/p50	6 h, 4 d, 10 d	27
Cohort 3 Western blot (inflammatory/angiogenic markers)	Western blot	TNF-α, IL-17, VEGF, TG2, NF-κB p65	day 7	18
Cohort 4 Gene expression analysis	RT-qPCR	TNF-α, IL-17A, VEGF, cPLA_2_, RELA, TG2	day 3	18

**Table 3:** Nucleotide sequence of primers.

Protein	Gene mRNA	Forward (5′–3′)	Reverse (5′–3′)
TNF-α	*TNFA*	CTTCTCCTTCCTGATCGTGG	GCTGGTTATCTCTCAGCTCCA
IL-17A	*IL17A*	CGGACTGTGATGGTCAACCTGA	GCACTTTGCCTCCCAGATCACA
VEGF	*VEGFA*	AGGCACAGACATAGGAGAGA	TTTCCCTTTCCTCGAACTGA
cPLA_2_	*PLA2G4A*	CATGCCCGTAATACCAGCAC	GCAAACAAGATGAATGGGAAC
ACTB	*β-actin*	TGTCACCAACTGGGACGATA	GGGGTGTTGAAGGTCTCAAA
NF-κB p65	*RELA*	AGGCAAGGAATAATGCTGCTG	ATCATTCTCTAGTGTCTGGTTGG

## Data Availability

The data supporting the findings of this study are available from the corresponding author upon reasonable request.

## Supplementary Materials

The following supporting information can be downloaded at: https://www.mdpi.com/article/10.3390/ijms27073247/s1.

## Abbreviations

The following abbreviations are used in this manuscript:

ACTB	Beta-actin gene
ANOVA	Analysis of Variance
cPLA_2_	Cytosolic Phospholipase A_2_
CNV	Corneal Neovascularization
ECL	Enhanced Chemiluminescence
FP	Fusion Protein (Cementoin-SLPI)
H&E	Hematoxylin and Eosin
IL-17	Interleukin-17
NF-κB	Nuclear Factor kappa B
OCT	Optimal Cutting Temperature
PVDF	Polyvinylidene Difluoride
ROI	Region of Interest
RT-qPCR	Reverse Transcription quantitative Polymerase Chain Reaction
SEM	Standard Error of the Mean
SLPI	Secretory Leukocyte Protease Inhibitor
TG2	Transglutaminase 2
TNF-α	Tumor Necrosis Factor alpha
VEGF	Vascular Endothelial Growth Factor
