# Supplementary material for: Downregulation of the Transglutaminase 2–NF-κB Inflammatory Axis by a Fusion Protein of Cementoin and Secretory Leukocyte Protease Inhibitor Reduces Corneal Angiogenesis
Source: Int J Mol Sci. 2026 Apr 2;27(7):3247. doi: 10.3390/ijms27073247 (PMC13073199; doi:10.3390/ijms27073247)
Supplement: Supplementary file 1 [file ijms-27-03247-s001.zip › ijms-4187175-supplementary.pdf]

International Journal of
Molecular Sciences

MDPI

Article

Downregulation of the TG2-NFkB inflammatory axis by a fusion protein of Cementoin-SLPI reduces corneal angiogenesis.

Juan Pablo Salica [1,2], Constanza Potilinski[1], Gustavo Ortiz[1], Paulo Maffia[3], Diego Guerrieri[3], Eduardo Chuluyan[3,4] and Juan Eduardo Gallo [1,2]*

SUPPLEMENTARY MATERIAL

Table S1. Nonparametric statistical analysis of residual epithelial defect area at 16 h post-injury.

Comparison	Mean Rank Difference	Adjusted p Value	Significance
Buffer vs SLPI	1.50	>0.9999	Ns
Buffer vs FP	5.50	0.2231	Ns
SLPI vs FP	4.00	0.5831	Ns

Values represent adjusted p-values from Dunn's multiple comparisons test following Kruskal–Wallis analysis ($\alpha = 0.05$).

Table S2. Kruskal–Wallis analysis of corneal opacity scores at day 7 post-alkali injury

Group	Median	25th–75th Percentile (IQR)	Mean ± SD	n
Buffer	4.0	3.0–4.0	3.667 ± 0.516	6
SLPI	2.5	1.75–4.0	2.667 ± 1.211	6
FP	2.0	1.0–2.5	2.000 ± 1.095	6
Healthy	0.0	0.0–0.0	0.000 ± 0.000	6

Kruskal–Wallis test: H = 16.96; p = 0.0007 (approximate)

Table S3. One-way ANOVA with Tukey's multiple comparisons test for TNF-α protein expression levels.

**Comparison**	**Mean Difference**	**95% CI of Difference**	**Adjusted p Value**	**Significance**
FP vs. SLPI	−0.6119	−0.9347 to −0.2890	0.0005	***
FP vs. Buffer	−0.5162	−0.8390 to −0.1933	0.0023	**
SLPI vs. Buffer	0.09568	−0.2272 to 0.4185	0.7266	ns

Data represent densitometric quantification of TNF-α protein levels normalized to loading control.

Table S4. One-way ANOVA and Tukey's multiple comparisons test for IL-17 protein expression following alkali injury

Quantitative densitometric analysis of IL-17 protein levels in corneal tissue (n = 6 per group). Statistical analysis was performed using one-way ANOVA followed by Tukey's post hoc test. Significance was set at $p < 0.05$.

Comparison	Mean Difference	95% CI of Difference	Adjusted p Value	Significance
FP vs. SLPI	-0.2844	-0.4822 to -0.0866	0.0053	**
FP vs. Buffer	-0.2710	-0.4688 to -0.0732	0.0076	**
SLPI vs. Buffer	0.0134	-0.1844 to 0.2112	0.9831	ns

Data represent densitometric quantification of IL-17 protein levels normalized to loading control.

Table S5. One-way ANOVA with Tukey's multiple comparisons test for VEGF protein expression levels

One-way ANOVA revealed significant differences among groups (α = 0.05). Post hoc comparisons were performed using Tukey's multiple comparisons test (n = 6 per group).

**Comparison**	**Mean Difference**	**95% CI of Difference**	**Adjusted p Value**	**Significance**
FP vs. SLPI	−0.6632	−0.9462 to −0.3802	<0.0001	****
FP vs. Buffer	−0.4757	−0.7588 to −0.1927	0.0015	**
SLPI vs. Buffer	0.1875	−0.0956 to 0.4705	0.2301	ns

Data represent densitometric quantification of VEGF protein levels normalized to loading control.

Table S6. One-way ANOVA with Tukey's multiple comparisons test for TG2 protein expression levels

One-way ANOVA revealed significant differences among groups (α = 0.05). Post hoc comparisons were performed using Tukey's multiple comparisons test (n = 6 per group).

Comparison	Mean Difference	95% CI of Difference	Adjusted p Value	Significance
Buffer vs. SLPI	0.2454	0.1390 to 0.3518	<0.0001	****
Buffer vs. FP	0.3767	0.2703 to 0.4831	<0.0001	****
SLPI vs. FP	0.1313	0.02487 to 0.2377	0.0154	*

Data represent densitometric quantification of TG2 protein levels normalized to loading control.

Table S7. One-way ANOVA with Tukey's multiple comparisons test for NF-κB p65 protein expression levels

One-way ANOVA revealed significant differences among groups (α = 0.05). Post hoc comparisons were performed using Tukey's multiple comparisons test (n = 6 per group).

Comparison	Mean Difference	95% CI of Difference	Adjusted p Value	Significance
Buffer vs. SLPI	0.2021	0.06628 to 0.3379	0.0041	**
Buffer vs. FP	0.3779	0.2421 to 0.5137	<0.0001	****
SLPI vs. FP	0.1758	0.03999 to 0.3116	0.0112	*

Data represent densitometric quantification of NF-κB p65 protein levels normalized to loading control.

Figure S1. Digital image-based quantification of corneal neovascularization.

A

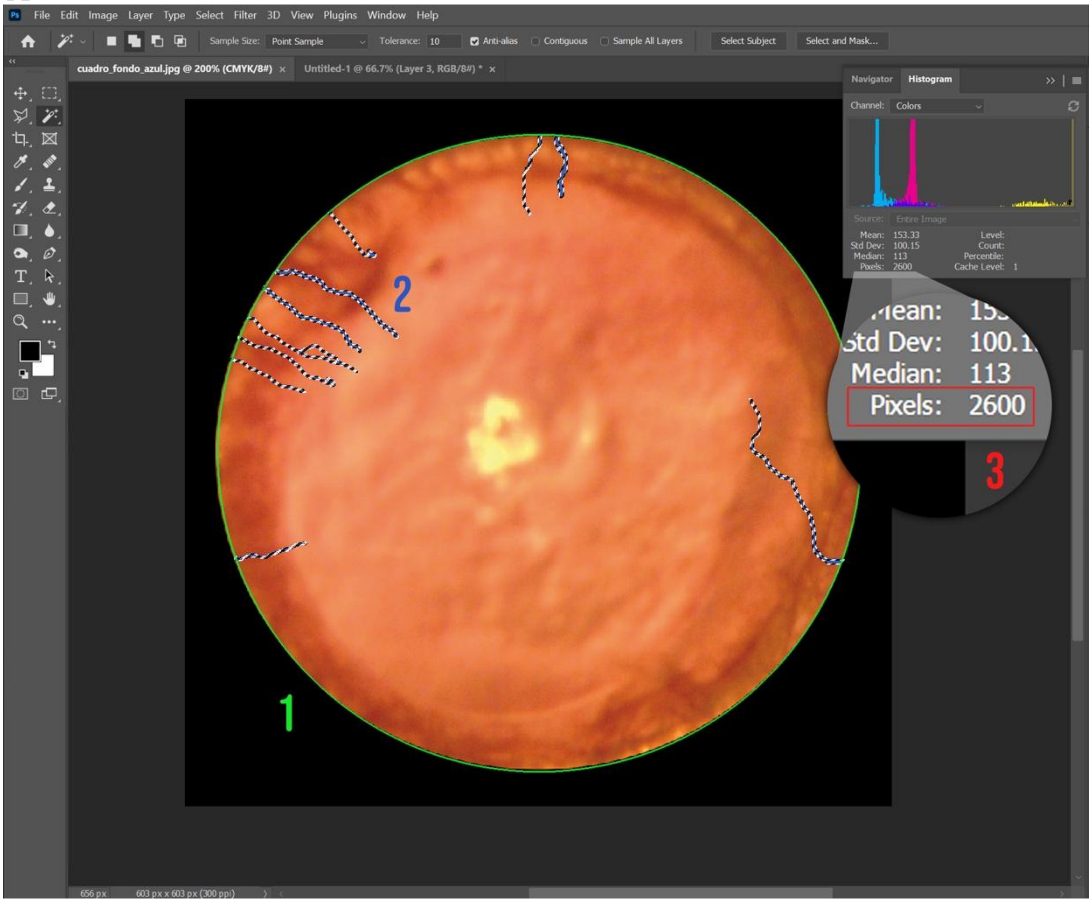

A) Representative example of the image-processing workflow used for corneal neovascularization analysis.

(1) A standardized circular region of interest (ROI) was aligned with the sclerocorneal limbus to normalize corneal size and resolution across samples.

(2) Corneal blood vessels were manually traced in blue using high magnification.

(3) The vascularized area was quantified by pixel counting of the traced region, obtained directly from the image information panel. Neovascularization was expressed as the percentage of the vascularized area relative to the total corneal area.

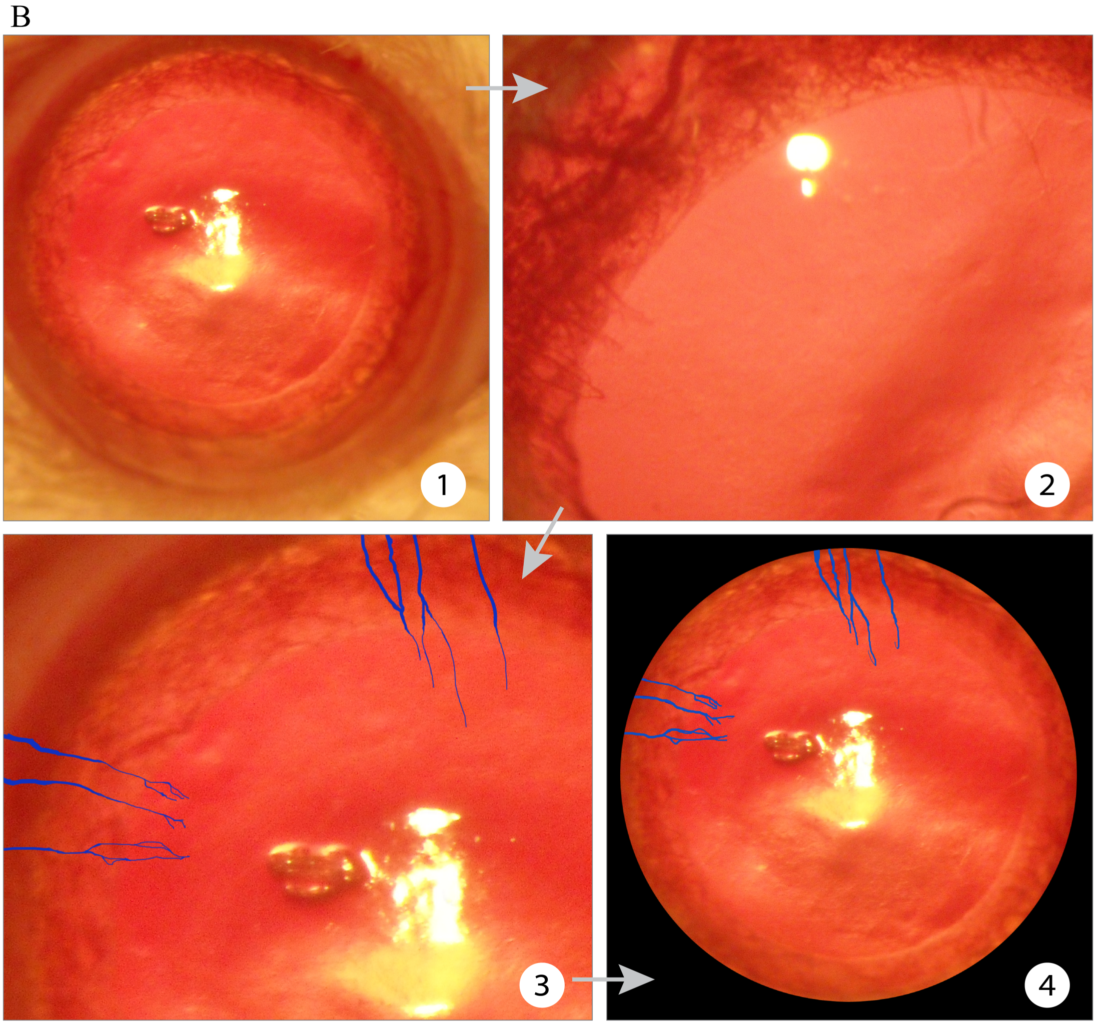

B) Representative images illustrating the use of an oblique view to support corneal neovascularization assessment.

(1, 3, 4) Frontal views of the cornea.

(2) Oblique view showing the same corneal region, used to confirm the corneal origin and direction of a neo-vessel when its identification was uncertain in the frontal image, particularly in the peripheral cornea.

Figure S2. Representative slit-lamp examination of corneal opacity following alkali injury.

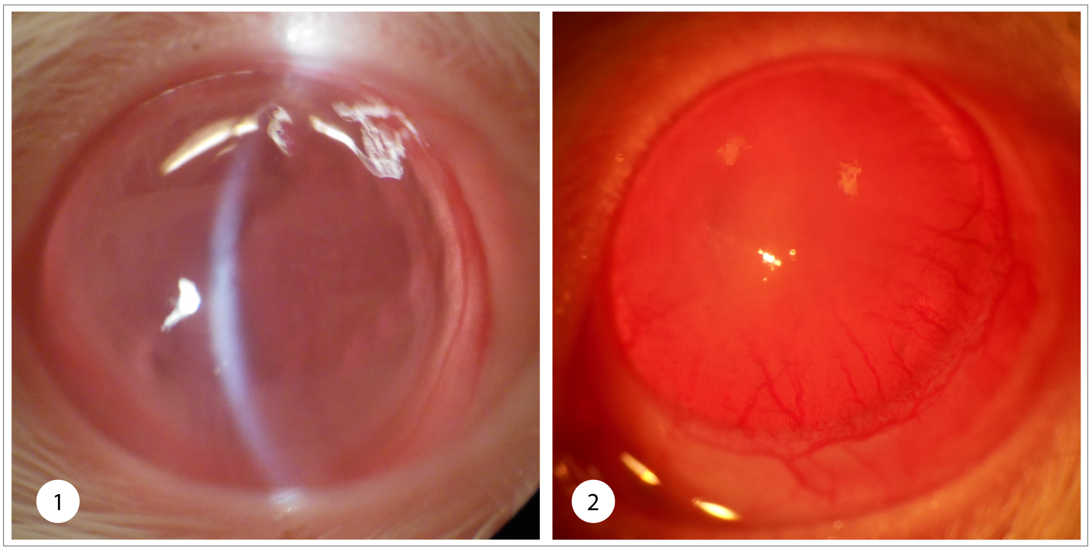

Representative slit-lamp biomicroscopy images illustrating the assessment of corneal opacity on day 7 post-injury. Frontal illumination allows evaluation of overall corneal transparency and haze, while oblique illumination enhances visualization of stromal light scatter and subtle opacity changes. The oblique view provides complementary information, particularly for detecting mild to moderate stromal haze that may not be readily apparent under direct illumination. These images exemplify the grading criteria applied across experimental groups.
